# Multimodal Radiogenomic Imaging in Oropharyngeal Squamous Cell Carcinoma: Implications for Dentomaxillofacial Radiology

**DOI:** 10.3390/medsci14020174

**Published:** 2026-03-31

**Authors:** Elaine Dinardi Barioni, Kaan Orhan, Ana Cristina Borges-Oliveira, Sérgio Lúcio Pereira de Castro Lopes, Andre Luiz Ferreira Costa

**Affiliations:** 1 Postgraduate Program in Dentistry, Dentomaxillofacial Radiology and Imaging Laboratory, Cruzeiro do Sul University (UNICSUL), São Paulo 01506-000, Brazil; elainedinardi2@gmail.com; 2 Department of Dentomaxillofacial Radiology, Faculty of Dentistry, Ankara University, Ankara 06560, Turkey; kaan.orhan@dentistry.ankara.edu.tr; 3 School of Dentistry, Department of Social and Preventive Dentistry, Universidade Federal de Minas Gerais, Belo Horizonte 31270-901, Brazil; anacboliveira@ufmg.br; 4 Department of Diagnosis and Surgery, Institute of Sciences and Technology, São Paulo State University (UNESP), São José dos Campos 12245-000, Brazil; sergio.lopes@unesp.br; 5 Department of Anesthesiology, Oncology and Radiology, Faculty of Medical Sciences, University of Campinas (UNICAMP), Campinas 13083-887, Brazil

**Keywords:** artificial intelligence, diagnostic imaging, HPV prediction, oncology, precision imaging

## Abstract

Radiogenomics examines associations between imaging phenotypes and underlying biological characteristics across cancer types. This structured narrative review focuses on oropharyngeal squamous cell carcinoma (OPSCC) and evaluates how genomic programs characteristic of HPV-positive and HPV-negative tumors have been investigated across computed tomography (CT), magnetic resonance imaging (MRI) and positron emission tomography/computed tomography (PET/CT) as variations in heterogeneity, diffusion patterns, perfusion and metabolic activity. A structured literature search was conducted in PubMed/MEDLINE, Scopus and Web of Science to identify studies on radiomics and radiogenomics in OPSCC and related head and neck cancers. After screening and eligibility assessment, 81 studies were included in the narrative synthesis. The reviewed literature indicates that imaging-derived features have been associated with HPV status, hypoxia-related signatures, extranodal extension and treatment outcomes. However, the current evidence base remains heterogeneous and is largely composed of retrospective, single-institution studies with relatively small cohorts. Methodological challenges, including variability in imaging acquisition, segmentation and feature harmonization, limit reproducibility and generalizability. Although cone-beam computed tomography (CBCT) is not used for primary OPSCC staging and no CBCT-based radiogenomic studies in OPSCC have been reported, existing radiomics research in dentomaxillofacial imaging suggests its potential as a hypothesis-generating modality for future investigation. Overall, current evidence supports the biological plausibility of radiogenomic imaging signatures in OPSCC, while emphasizing the need for larger multicenter datasets, standardized imaging protocols and prospective validation before clinical implementation.

## 1. Introduction

Oropharyngeal squamous cell carcinoma (OPSCC) encompasses two biologically distinct disease entities whose differences profoundly influence prognosis, treatment responsiveness and metastatic behavior [1]. Human papillomavirus (HPV)–positive tumors characteristically demonstrate genomic stability, immune activation and enhanced radiosensitivity, whereas HPV-negative tumors evolve through carcinogen-driven pathways marked by TP53 dysfunction, chromosomal instability, hypoxia and aggressive invasive potential [2,3]. These divergent biological trajectories have been firmly established in contemporary oncologic literature and translate into clinically relevant outcome disparities [1,4]. Recent imaging-driven investigations have further reinforced that HPV-positive and HPV-negative tumors not only behave differently but also appear fundamentally different on cross-sectional imaging, reflecting their underlying biological architecture [5,6,7,8].

Although these biological differences are well established, conventional radiology primarily captures their macroscopic manifestations. However, qualitative interpretation alone is insufficient to resolve the complexity of the molecular programs driving OPSCC progression. Radiogenomics is an integrative approach linking quantitative imaging features to genomic, transcriptomic and microenvironmental signatures, offers a biologically informed extension to traditional imaging assessment [9,10,11]. By extracting multidimensional features from computed tomography (CT), magnetic resonance imaging (MRI) and positron emission tomography combined with computed tomography (PET/CT), radiogenomics enables non-invasive characterization of tumor heterogeneity, cellular organization, metabolic gradients and stromal dynamics [9,11,12]. This quantitative phenotyping has been investigated as a means of predicting HPV status, hypoxia, epithelial–mesenchymal transition, radiosensitivity and the risk of nodal metastasis [13,14,15].

Despite substantial advances in radiogenomics within general head and neck oncology, its implications for dentomaxillofacial radiology remain comparatively underexplored. OPSCC frequently involves the base of the tongue, tonsillar complex, parapharyngeal spaces and cervical lymph nodes [16,17], anatomical regions routinely encompassed in dentomaxillofacial imaging examinations. As dentomaxillofacial radiologists increasingly participate in multidisciplinary diagnostic pathways, there is growing interest in imaging approaches that can complement morphological assessment with biologically informed insights. In this context, Cone-Beam Computed Tomography (CBCT), traditionally used for dentoalveolar evaluation, is also evolving through radiomic adaptation and may eventually contribute to the characterization of osseous involvement or incidental soft-tissue abnormalities relevant to OPSCC.

From a dentomaxillofacial radiology perspective, the relevance of OPSCC radiogenomics becomes particularly evident. Dentomaxillofacial radiologists frequently interpret imaging studies that encompass the oropharyngeal region—including the base of tongue, tonsillar complex, parapharyngeal spaces and cervical lymph nodes—either in dedicated head and neck examinations or as part of broader dentomaxillofacial imaging protocols [16,17]. Consequently, they may encounter incidental oropharyngeal abnormalities and participate in the early recognition or referral pathway of patients later diagnosed with OPSCC. In this context, the ability to interpret imaging findings not only as structural abnormalities but also as potential indicators of underlying tumor biology becomes increasingly relevant. Radiogenomics therefore provides a biologically informed interpretive perspective that links multimodal imaging phenotypes to molecular programs associated with HPV status, hypoxia and tumor aggressiveness [9,10,11,13,14,15], potentially enhancing the diagnostic and consultative role of dentomaxillofacial radiologists within multidisciplinary care.

The originality of this review lies in synthesizing radiogenomic mechanisms, multimodal imaging correlates and clinically oriented applications specifically through the lens of dentomaxillofacial radiology. Previous reviews have examined radiomics and radiogenomics in head and neck oncology from broader oncologic imaging or artificial intelligence perspectives (e.g., Bruixola et al. [18]. Chen et al. [9], and Saxena et al. [19]). In contrast, the present review focuses specifically on OPSCC and examines how radiogenomic imaging findings derived primarily from CT, MRI and PET/CT may inform interpretation and clinical reasoning within dentomaxillofacial radiology practice. By integrating multimodal radiomics, genomic determinants within this specialty context, the review provides a critical synthesis of current evidence while delineating established knowledge, emerging concepts and unresolved gaps that define priorities for future research.

Accordingly, this review does not propose CBCT-based radiogenomics as an established application in OPSCC. Rather, it examines current radiogenomic evidence derived from CT, MRI and PET/CT while identifying research directions that may become relevant to dentomaxillofacial radiology. In particular, the review highlights CBCT as a currently unexplored imaging platform for radiogenomic investigation in OPSCC, emphasizing that its potential role remains hypothetical and requires future validation in molecularly annotated datasets.

## 2. Methods (Literature Search Strategy)

This study was conducted as a structured narrative review. A literature search was conducted to identify studies evaluating radiomics and radiogenomics in OPSCC, with particular emphasis on imaging modalities relevant to head and neck and dentomaxillofacial radiology. Searches were performed in PubMed/MEDLINE, Scopus and Web of Science, covering the period from January 2008 through October 2025 in each database. The search strategy combined controlled vocabulary and free-text terms related to quantitative imaging, multi-omics modeling and molecularly driven disease characterization, reflecting the inherently multidisciplinary nature of radiogenomics. Search terms included radiomics, texture analysis, radiogenomics, multi-omics, machine learning, deep learning, CT, MRI, PET/CT, CBCT imaging and HPV-related OPSCC, as well as molecular correlates such as immune activation, hypoxia, epithelial–mesenchymal transition (EMT) signatures and transcriptional subtypes. An example search strategy included combinations such as (“radiomics” OR “radiogenomics” OR “texture analysis”) AND (“OPSCC” OR “oropharyngeal cancer” OR “head and neck cancer” OR “head and neck squamous cell carcinoma”) AND (“CT” OR “MRI” OR “PET/CT” OR “CBCT”) AND (“HPV” OR “hypoxia” OR “immune”). This broad approach aligns with established methodological expectations for imaging-based artificial intelligence research and narrative syntheses in radiology.

All retrieved titles and abstracts were assessed. Screening was performed by reviewing titles and abstracts for relevance to imaging biomarkers and radiogenomic associations in OPSCC, followed by full-text evaluation of eligible studies. Disagreements in study inclusion were resolved through consensus. Articles were included when they provided original peer-reviewed quantitative imaging analyses evaluating associations between imaging features and biological, molecular or clinically relevant outcomes related to OPSCC. Because radiogenomic investigations specifically dedicated to OPSCC remain relatively limited, studies focusing on head and neck squamous cell carcinoma (HNSCC) were also considered when their imaging–biology relationships were directly applicable to OPSCC, based on the principle of biological transferability. This included studies addressing HPV-related pathways, hypoxia signatures, immune microenvironment features or methodological developments relevant to OPSCC imaging. Although this approach may introduce selection bias, it was adopted to avoid excluding biologically informative and methodologically robust evidence when OPSCC-specific data were limited.

Studies were excluded when they lacked full text, did not involve quantitative feature extraction, or did not evaluate relationships between imaging features and biological or clinical endpoints relevant to OPSCC. Studies restricted to purely technical imaging descriptions without analyzable radiomic or molecular outcomes were also excluded. In addition, reference lists of all eligible publications were manually screened to ensure comprehensive coverage of the domain.

To improve transparency regarding study selection, the literature screening process followed a structured four-stage framework summarized in Table 1.

For each included study, methodological details were extracted with attention to imaging protocols, segmentation procedures, feature extraction workflows and validation strategies. Radiomic feature extraction was interpreted within established frameworks describing first-order statistics, morphological descriptors and higher-order textural matrices, as well as wavelet- and filter-based feature families. The reliability and stability of these measures were evaluated based on existing radiomics standards and prior demonstrations of feature robustness and variability.

Because radiogenomic studies differ in their analytical pipelines, particular attention was given to how imaging features were linked to molecular data. Studies incorporating transcriptomic signatures, immune microenvironment profiles, HPV-related biological markers or habitat-based subregional phenotyping were examined regarding feature selection strategies, harmonization procedures and model validation. Multimodal and AI-driven analytical strategies were interpreted in the context of recent integrative radiogenomic approaches combining imaging, clinical and genomic inputs to improve biological characterization and prognostic stratification.

Following these procedures, 81 studies met the eligibility criteria and were included in the narrative synthesis, representing the current spectrum of evidence in OPSCC radiomics and radiogenomics, from foundational quantitative imaging methodologies to emerging multimodal and AI-enhanced analytical models.

Because this study follows a structured narrative review design aimed at conceptual synthesis rather than quantitative comparison, a formal risk-of-bias assessment was not performed. Instead, studies were qualitatively evaluated based on methodological clarity, relevance to OPSCC, and reporting of imaging and analytical pipelines.

## 3. Biological Divergence Between HPV-Positive and HPV-Negative OPSCC

The biological divergence between HPV-positive and HPV-negative OPSCC has been extensively described in the Introduction and in prior literature [20,21], and is therefore not repeated in detail here. This section focuses on how these biological differences have been investigated through imaging and radiogenomic associations.

Several imaging studies have reported that HPV-positive tumors tend to display smoother margins, more organized enhancement patterns, reduced textural complexity, and more cohesive apparent diffusion coefficient (ADC) distributions on MRI [22,23,24]. It is important to emphasize that these imaging–genomic relationships represent statistical associations observed across patient cohorts rather than direct molecular measurements at the individual tumor level. These imaging features have been associated with immune-active tumor microenvironments and relatively homogeneous tumor architecture. However, these relationships remain influenced by imaging protocols, cohort characteristics, and segmentation approaches used in radiomic studies [22,25].

In contrast, HPV-negative tumors frequently demonstrate imaging patterns associated with greater biological heterogeneity. Corresponding studies describe irregular tumor borders, heterogeneous enhancement, necrotic compartments, increased textural entropy, variable ADC maps, and pronounced metabolic gradients on PET/CT [7,8,12,22,23]. These phenotypes have been reported to be associated with genomic instability, hypoxia-related pathways, and epithelial–mesenchymal transition (EMT), although the strength of these associations varies across datasets and analytical methodologies.

Taken together, these biological and imaging patterns suggest the presence of distinct radiogenomic profiles in HPV-positive and HPV-negative OPSCC [2,3,26]. While some associations, particularly those related to HPV status and imaging phenotype, have been reported consistently across independent cohorts, others remain exploratory and require validation in larger multicenter radiogenomic datasets.

## 4. Genomic Determinants and Their Imaging Correlates

Several genomic alterations have been reported to influence imaging phenotypes in OPSCC, although the strength and reproducibility of these associations vary across studies and methodological designs. Furthermore, many of these associations are derived from radiomics-based analyses rather than fully integrated radiogenomic studies incorporating matched molecular data and should therefore be interpreted accordingly.

Alterations in PIK3CA, frequently reported in HPV-positive disease, have been associated with more uniform vascular patterns and smoother radiomic texture profiles in several radiomic analyses [27]. TP53 mutations, which dominate the genomic landscape of HPV-negative OPSCC, have been linked to imaging patterns characterized by greater morphological heterogeneity and irregular stromal interfaces in several retrospective imaging analyses [7,8,22,23], and increased textural entropy in CT and MRI. FAT1 and NOTCH1 alterations have been associated with disordered growth patterns, EMT activation and stromal remodeling, which may manifest radiologically as infiltrative tumor margins and heterogeneous imaging patterns in some radiomics studies [7,22,26,28]. TERT promoter alterations, strongly associated with metabolic aggressiveness, frequently appear as elevated FDG uptake heterogeneity and prominent glycolytic gradients on PET/CT [12,26].

It is important to note that most reported genotype–phenotype associations in OPSCC are derived from retrospective radiomics studies with heterogeneous study designs, cohort sizes, and analytical pipelines. As a result, effect sizes are not directly comparable across studies, and external validation remains limited.

The most reported genotype–phenotype correlations in OPSCC derive from retrospective radiomics studies with heterogeneous imaging protocols, cohort sizes and analytical pipelines [10,12,18,29]. Only a limited number of investigations include external validation cohorts or multicenter datasets, and effect sizes are often not directly comparable across studies due to differences in feature extraction, feature selection and modeling strategies. Consequently, many imaging–genomic relationships should be interpreted as emerging associations rather than fully validated biomarkers [10,18,29].

The major genomic subtypes of OPSCC and their characteristic imaging phenotypes are summarized in Table 2 [1,2,3,7,8,12,22,26,30,31,32].

Hypoxia represents another biologically and radiologically relevant axis. Tumors enriched with HIF1A, CA9 and VEGFA signatures often demonstrate disrupted perfusion, low-ADC nests and heterogeneous contrast enhancement [11,26]. Conversely, immune activation and antigen presentation pathways observed in HPV-positive disease produce more organized vascular patterns, restricted textural variability and cohesive diffusion characteristics [2,8].

It is important to distinguish between radiomics studies that correlate imaging features with clinical outcomes and true radiogenomic investigations that directly integrate molecular datasets. In OPSCC, many reported associations remain radiomic correlations rather than fully integrated radiogenomic models. Radiogenomic findings should therefore be interpreted as statistical associations observed across patient cohorts rather than direct molecular measurements at the individual tumor level.

Taken together, these reported associations suggest biologically plausible links between genomic programs and imaging phenotypes in OPSCC, although further validation in prospective and multicenter radiogenomic studies remains necessary [2,7,8,26,28,30]. These genomic–imaging relationships provide a biological explanation for radiomic signatures observed across OPSCC and underpin the construction of predictive models that integrate CT, MRI and PET/CT with molecular datasets. The strength and type of evidence supporting these associations, including whether they derive from retrospective radiomics analyses or integrated radiogenomic studies, are summarized in the accompanying Table 3 [2,7,8,12,23,26,28,30].

**Figure 1:**
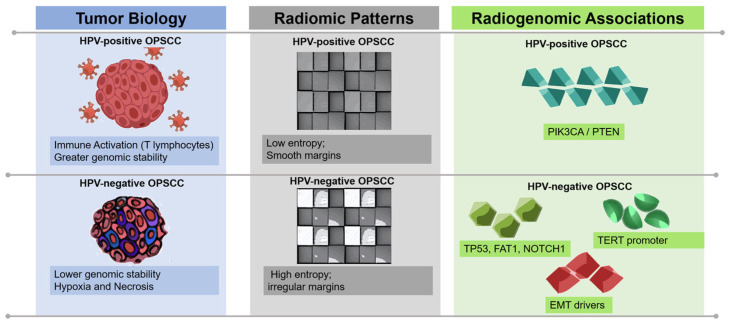
Integrated radiogenomic ecosystem of OPSCC. Biological programs shape multimodal imaging phenotypes across CT, MRI, PET/CT and CBCT. These signatures have been reported to correlate with underlying tumor behavior and support key radiogenomic predictions, including HPV status, aggressiveness and treatment response.

These biological programs, their corresponding imaging signatures and associated clinical predictions are summarized visually in Figure 1, which illustrates the integrated radiogenomic ecosystem of OPSCC.

To provide a structured overview of the literature discussed in this review, Table 4 summarizes representative radiomic and radiogenomic studies in OPSCC according to imaging modality, analytical approach, genomic targets and primary clinical outcomes. Detailed characteristics of representative studies, including cohort size, study design and analytical methods, are provided in Supplementary Table S1.

However, reproducibility across institutions and imaging protocols remains a significant challenge, and many reported associations require validation in larger, multicenter radiogenomic datasets.

## 5. Multimodal Imaging in OPSCC Radiogenomics

The roles of CT, MRI, PET/CT and CBCT in OPSCC imaging, including their clinical applications and corresponding levels of evidence, are summarized in Table 5 [7,8,12,14,22,23,24,25,26,28,45,46,47,48,49,50,51,52,53].

Although CT, MRI and PET/CT each contribute valuable radiomic features for OPSCC characterization, their respective methodological limitations should also be acknowledged. CT-based radiomics is highly dependent on reconstruction algorithms and voxel resolution, which can influence texture reproducibility. MRI-derived features may vary substantially depending on sequence selection and field strength, particularly for diffusion-weighted imaging. PET/CT radiomics, while sensitive to metabolic heterogeneity, can be affected by partial-volume effects and acquisition protocol variability. Consequently, multimodal integration has been proposed as a strategy to mitigate modality-specific limitations and improve biological inference in OPSCC radiogenomic modeling [10,12,18]. These modality-specific limitations may influence clinical interpretation. CT-based features are generally more reproducible across institutions but may lack sensitivity to microstructural changes. MRI provides superior characterization of tissue microenvironment but is more sensitive to acquisition variability. PET/CT offers functional metabolic information but is influenced by spatial resolution and partial-volume effects. These differences highlight the complementary nature of multimodal imaging in OPSCC radiogenomics [10,12,18,22,23,24,34,36,54,55].

Reported predictive performances across radiomics studies in OPSCC typically fall within moderate-to-high discriminative ranges, with several investigations reporting area-under-the-curve values between approximately 0.70 and 0.90 depending on modality, feature selection strategy and cohort characteristics. However, direct comparison across studies remains challenging because of heterogeneity in segmentation approaches, feature harmonization strategies and validation protocols [10,25,33,37].

### 5.1. CT Phenotypes and Biological Associations

Contrast-enhanced CT remains the most widely used imaging modality in OPSCC evaluation, serving as the primary source for radiomic feature extraction. Its spatial resolution and consistency across institutions lend themselves to robust texture analysis. Features such as entropy, gray-level non-uniformity, and run-length variability have shown strong associations with genomic instability, hypoxia-related gene expression, and aggressive stromal remodeling. Necrotic components within primary tumors and lymph nodes have frequently been associated with hypoxia-driven metabolic reprogramming and transcriptional programs linked to poor outcomes [33,34,37,55].

### 5.2. MRI and Microstructural Biomarkers

Multiparametric MRI offers a biologically rich complement to CT. Diffusion-weighted imaging and ADC maps are particularly informative, as they reflect microstructural organization, cellular density, and stromal architecture. HPV-positive tumors have frequently been reported to demonstrate relatively uniform ADC distributions, which have been associated with cohesive tumor architecture and immune-rich microenvironments. In contrast, HPV-negative tumors often exhibit greater ADC heterogeneity, which has been linked in several imaging studies to proliferative and hypoxia-related tumor regions. Dynamic contrast-enhanced MRI can further highlight perfusion deficits and angiogenic pathways associated with EMT activation and metabolic stress. These MRI-derived signatures contribute significantly to HPV status prediction and treatment-response modeling [2,14,22,23,24,26].

### 5.3. PET/CT and Metabolic Signatures

PET radiomic signatures have been reported to correlate with metabolic programs linked to oxidative stress pathways, TERT activation and tumor stemness features. Tumors with high metabolic heterogeneity frequently exhibit poor outcomes and aggressive genomic profiles. PET/CT thus acts as a critical component of multimodal radiogenomic modeling, particularly in HPV-negative disease [12,25,26,44].

### 5.4. The Position of CBCT in Radiogenomic Approaches

Compared with CT, MRI and PET/CT, the evidence base for CBCT in OPSCC radiomics or radiogenomics remains substantially more limited. Accordingly, the following discussion is presented as a translational and exploratory perspective rather than a summary of established radiogenomic evidence.

Although CBCT is not routinely used for the primary staging of OPSCC, it represents a foundational modality in dentomaxillofacial imaging. Incidental findings on CBCT, such as asymmetric soft-tissue volumes, base-of-tongue irregularities or suspicious cervical nodes, may serve as early indicators of oropharyngeal pathology and can precede formal head and neck imaging [45,46,47].

Radiomics applications in dentomaxillofacial CBCT have been investigated in a variety of osseous and periapical conditions, demonstrating the feasibility of extracting reproducible texture-based biomarkers under controlled imaging conditions [48,49,50,51,52,53,56]. It should be acknowledged that several of these studies originate from the authors’ research group [49,50,53,57] and are cited here primarily to illustrate the technical feasibility of CBCT radiomics pipelines, rather than to imply the existence of OPSCC-specific radiogenomic evidence.

Evidence from other oncologic imaging domains provides methodological precedents for radiogenomic integration. CBCT-based delta-radiomics has been explored in prostate radiotherapy workflows, and contrast-enhanced cone-beam breast CT has demonstrated associations between imaging features and molecular subtypes. While these findings are not directly transferable to OPSCC, they illustrate how cone-beam imaging platforms may support radiogenomic investigations when combined with molecular annotation [58,59], but should be interpreted cautiously given differences in tumor biology, anatomical context and imaging protocols between disease sites.

Within dentomaxillofacial radiology, CBCT may therefore represent a potential platform for future radiogenomic research rather than an established application. Possible research directions include the investigation of bone invasion patterns, peri-tumoral remodeling and adjacent structural changes in relation to molecular features such as HPV status, TP53 alterations or hypoxia-related pathways. These hypotheses are supported by established radiogenomic findings in head and neck cancer, where imaging phenotypes have been associated with gene-expression signatures and tumor microenvironment characteristics [9,10,12,13,53,56,58,59].

Additionally, the routine availability of CBCT in dental practice introduces a potential pre-diagnostic window. Incidental CBCT examinations obtained prior to OPSCC diagnosis could be retrospectively analyzed for subtle imaging patterns that may be associated with subsequent tumor biology, including HPV status or hypoxia-related signatures [1,2,7,8,12,36,45].

Despite these conceptual possibilities, it is important to emphasize that CBCT-based radiogenomic applications in OPSCC remain unvalidated. Current evidence supports its role as a hypothesis-generating modality, and its integration into radiogenomic workflows will require molecularly annotated datasets, standardized acquisition protocols and prospective validation studies.

In this context, dentomaxillofacial radiologists can contribute to future developments by integrating CBCT findings with multimodal imaging and emerging radiogenomic knowledge, while recognizing that its current role remains exploratory.

## 6. Radiomics Pipeline and Methodological Considerations

Radiogenomic modeling is supported by a multi-step analytic process encompassing image acquisition, preprocessing, segmentation, feature extraction, feature selection and model development [33,34,42,54]. Each stage introduces potential sources of variability. These methodological challenges are particularly pronounced in OPSCC datasets because tumors arise within anatomically complex regions characterized by irregular soft-tissue interfaces, air cavities and frequent dental artifacts, which introduces additional variability in both image acquisition and segmentation. These factors can influence both segmentation accuracy and radiomic feature stability, especially in multicenter studies involving heterogeneous imaging protocols [34,36,54,55]. In OPSCC, heterogeneity in acquisition protocols, reconstruction kernels, voxel geometry and contrast timing can alter the distribution of radiomic features, complicating multi-institutional comparisons [34,36,54,55]. Preprocessing steps such as intensity normalization, voxel resampling, noise reduction and the co-registration of multimodal datasets are essential for reducing non-biological variability [9,60,61].

Segmentation remains a critical challenge in the anatomically complex oropharynx. Tumor boundaries often interface with air spaces, musculature and lymphoid tissue, and are frequently distorted by motion artifacts or dental hardware. Manual or semi-automatic segmentation by experienced radiologists offers reliability but is time-intensive and operator-dependent [62,63,64]. Comparative studies in head and neck radiomics have demonstrated that variations in segmentation strategy can significantly affect the distribution of extracted radiomic features and the stability of predictive models, underscoring the need for standardized annotation protocols, highlighting segmentation as a major source of variability [33,37]. Deep learning–based segmentation models promise increased consistency, although their performance in the presence of metallic artifacts or subtle tissue planes remains an area of active investigation [65].

The extraction of imaging features spans first-order intensity statistics, morphological descriptors, and second-order textural matrices derived from gray-level relationships [34,54]. Higher-order wavelet and transform-based features offer additional insight into subtle spatial patterns [66,67,68]. Because radiomics typically generates hundreds to thousands of features, dimensionality reduction and feature-selection strategies are required to identify stable, reproducible biomarkers [34,54,66,67,68]. Methods such as regularization techniques, information-theoretic selection and stability-based pruning improve model robustness. Harmonization techniques, including ComBat adjustments and deep-learning domain adaptation, have been proposed to mitigate scanner- and protocol-related variability across institutions [57]. In head and neck radiomics research, such approaches have been shown to improve feature stability and facilitate cross-cohort analyses when imaging protocols differ between centers, although their effectiveness remains dependent on consistent preprocessing pipelines and dataset characteristics [34,54].

These considerations are especially important for dentomaxillofacial radiologists who work within environments where metallic restorations, variable field-of-view selections and heterogeneous equipment parameters may influence radiomic feature stability [69].

Evidence from CBCT reproducibility studies further supports this concern. Even under controlled imaging conditions, only a subset of radiomic features remain stable across scanners, acquisition protocols, scatter levels, and motion amplitudes, indicating that CBCT can support radiomics analyses provided that protocol consistency is ensured and feature robustness is carefully evaluated [70].

While methodological guidelines propose standardized radiomics pipelines including harmonized acquisition parameters, reproducible segmentation strategies and external validation procedures, many current OPSCC studies only partially implement these recommendations. Consequently, differences between theoretical best practices and commonly applied workflows remain an important source of variability in radiogenomic investigations [34,54,57]. Standardization is therefore essential for meaningful integration of radiogenomics into clinical workflows [59].

**Figure 2:**
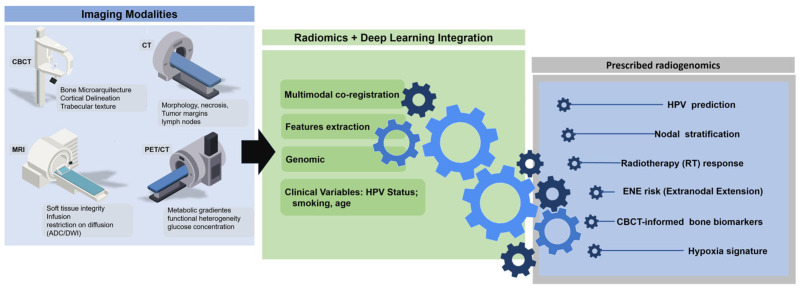
Radiomics workflow for OPSCC imaging. The analytic pipeline includes the preprocessing, segmentation and extraction of quantitative features that form the basis of downstream radiogenomic modeling.

Figure 2 summarizes the analytical workflow underlying radiomic modeling, from image acquisition to preprocessing, segmentation and feature engineering.

## 7. AI-Enhanced Radiogenomics and Multimodal Integration

Whereas radiomics quantifies imaging phenotypes, radiogenomics links these signatures to underlying molecular programs. AI-enhanced radiogenomics extends this approach by enabling the exploration of high-dimensional, non-linear relationships that may not be fully captured by handcrafted radiomic features, although these models remain sensitive to dataset size and methodological design [9,10,12].

The incorporation of AI into radiogenomic workflows has accelerated the identification of biologically meaningful imaging signatures [4,61]. Deep learning models can recognize complex spatial relationships that exceed the capabilities of handcrafted radiomic features, learning subtle patterns associated with immune activation, EMT, hypoxia and proliferative behavior [71,72,73]. When applied to CT, MRI and PET/CT datasets, several studies have reported improved predictive performance using deep learning models compared with traditional machine-learning pipelines, although quantitative comparisons remain heterogeneous across studies and are not directly comparable due to differences in dataset size, feature extraction and validation strategies [10,23,25]. A structured summary of these AI-based radiogenomic approaches is presented in Table 6 [10,11,12,13,14,15,23,25,34,37,42,54,71,72,74], which also distinguishes between approaches supported by existing OPSCC radiomics studies and those that remain exploratory or conceptual.

It should be noted that some AI strategies listed in Table 6 particularly graph neural networks and delta-radiomics approaches, remain largely conceptual in OPSCC radiogenomics and have not yet been validated in dedicated OPSCC datasets [18,19,75].

Despite these advances, the current evidence base remains constrained by methodological limitations. Many AI-driven radiogenomic models in OPSCC are developed using relatively small retrospective datasets, which increases the risk of overfitting and limits generalizability across institutions and imaging protocols. This limitation is particularly relevant in OPSCC radiogenomics, where cohort sizes are often limited and model performance may not generalize to independent datasets. External validation cohorts and prospective radiogenomic studies remain limited, highlighting the need for larger multicenter datasets to establish robust clinical performance [10,18,29].

Multimodal fusion represents a promising direction in radiogenomic research but also introduces additional methodological complexity. Integrating imaging, clinical variables and molecular datasets requires harmonization across heterogeneous data sources and careful management of missing or incomplete data. Variability in acquisition protocols, feature extraction pipelines and genomic annotation can significantly influence model performance and reproducibility [43,72]. For radiologists, these systems offer enhanced interpretability of subtle imaging findings and support the early identification of high-risk disease. Models that incorporate ADC maps, perfusion parameters, PET metabolic gradients and CT textural descriptors provide unified predictions that can directly influence multidisciplinary decision-making [8,22,23,25].

Explainable AI strategies are emerging to improve clinical acceptance by linking learned representations back to human-interpretable regions and biological processes [41]. However, current explainability methods often provide only partial insight into complex model behavior, and their integration into routine radiologic workflows remains an active area of research and currently limits the full clinical interpretability of AI-driven radiogenomic models in routine practice [76,77].

CBCT is intentionally not included in Table 6 because, despite its expanding radiomics literature in dentomaxillofacial applications, there is currently no OPSCC-specific radiogenomic evidence integrating CBCT with molecular signatures. Its role may evolve as annotated datasets become available.

**Figure 3:**
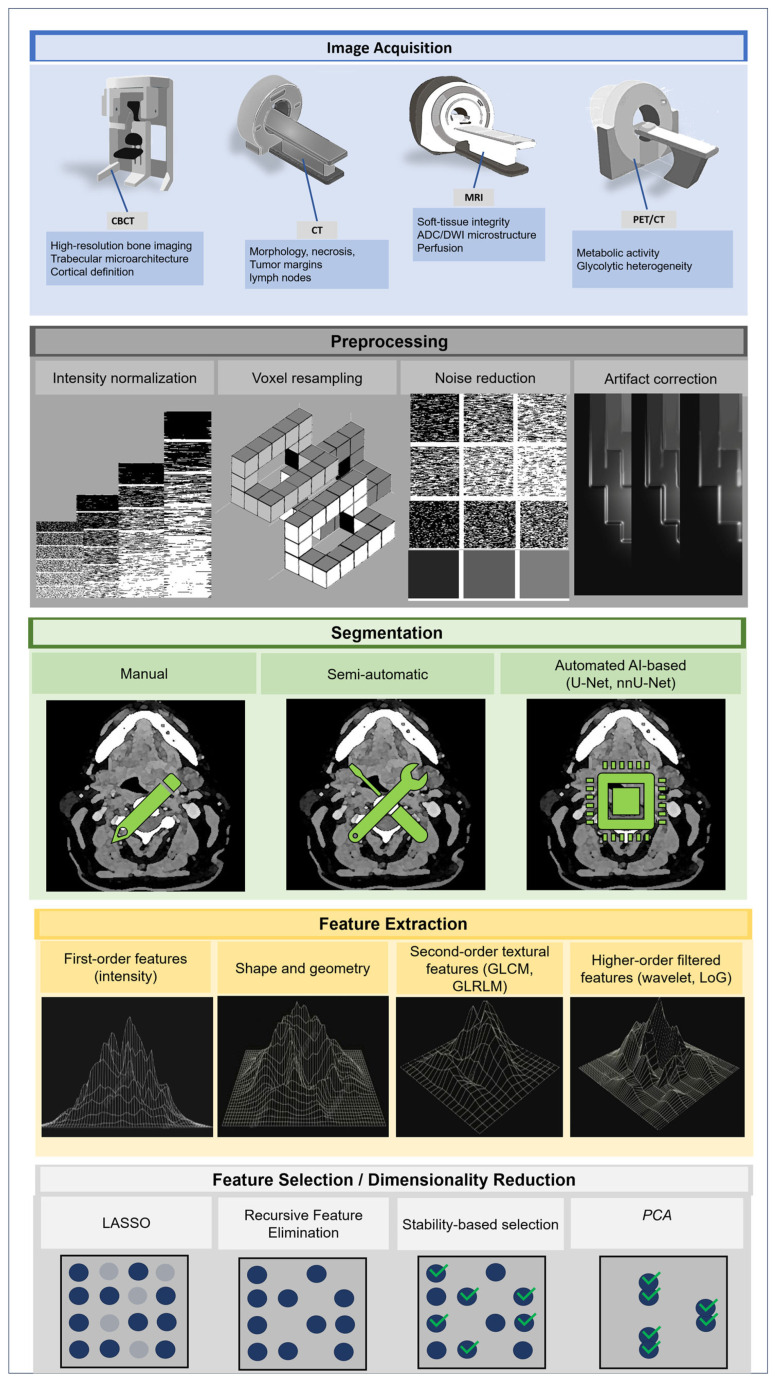
AI-integrated radiogenomic framework for OPSCC. Multimodal imaging features from CT, MRI, PET/CT and CBCT are combined with clinical and genomic information through AI-based models to generate biologically informed predictions.

As illustrated in Figure 3, multimodal radiomic features can be integrated with genomic, transcriptomic and clinical variables using AI-based fusion frameworks to generate biologically informed predictions.

In the context of dentomaxillofacial radiology, radiogenomics can be understood not merely as a predictive modeling strategy but as a biologically informed interpretive layer that enhances daily image evaluation. By linking multimodal imaging phenotypes to immune-rich, hypoxic, EMT-driven or genomically unstable tumor states, radiogenomics offers a framework for complementing traditional pattern recognition with biologically informed interpretation. This perspective enables dentomaxillofacial radiologists to read heterogeneity, perfusion abnormalities, ADC behavior and metabolic gradients as manifestations of underlying molecular programs, thereby enriching routine diagnostic reasoning and strengthening their contribution to multidisciplinary care.

## 8. Clinical Applications of Radiogenomics in OPSCC

Radiogenomics has been investigated as a promising approach for supporting radiologic evaluation of OPSCC, although most reported applications remain at the research stage. Prediction of HPV status represents one of the most extensively studied radiogenomic applications, with several radiomics investigations reporting associations between imaging phenotypes and HPV-related tumor biology [1,25,78]. However, most models have been developed in retrospective cohorts and require further multicenter validation before routine clinical implementation. When tissue sampling is limited or delayed, radiogenomic features can offer early biological insight that complements clinical examination [18,34]. In routine clinical practice, HPV status is reliably determined using p16 immunohistochemistry performed on diagnostic biopsy specimens and incorporated into American Joint Committee on Cancer (AJCC) staging. Consequently, imaging-based HPV prediction should currently be regarded primarily as a radiogenomic research application rather than a replacement for established pathological testing. The greatest potential clinical value of radiogenomics may instead lie in refining risk stratification within HPV-positive disease and identifying early treatment non-responders [1,25].

Radiogenomic features have also been explored as potential indicators of tumor aggressiveness [35,38]. Highly heterogeneous tumors identified on CT, MRI or PET/CT have been associated with hypoxic or genomically unstable phenotypes, although these correlations remain dependent on cohort characteristics and analytical methodology [11,35,36,38]. Radiomics-based analyses have also been investigated for improving the detection of extranodal extension and distinguishing reactive from metastatic cystic lymph nodes [6,79,80], though the reproducibility of these models across institutions remains under active investigation.

During and after therapy, radiogenomics helps identify patients at risk of residual disease or early recurrence. Delta-radiomic analyses that track imaging changes over time have been proposed to identify treatment-induced biological shifts that may not be visually apparent. However, longitudinal radiomics introduces additional methodological challenges, including the need for consistent acquisition protocols, standardized timing of follow-up imaging and harmonization of feature extraction across time points [18,75].

Overall, these studies illustrate the potential clinical relevance of radiogenomics in OPSCC, but most reported applications remain investigational and require further validation in prospective multicenter datasets before integration into routine clinical workflows [10,18,29].

A practical example may illustrate the potential clinical relevance of radiogenomic interpretation. In a patient with biopsy-confirmed HPV-positive OPSCC undergoing pre-treatment MRI and PET/CT, marked intratumoral heterogeneity, low-ADC subregions and pronounced metabolic gradients may be observed. While conventional imaging interpretation would primarily describe tumor extent and nodal status, radiogenomic-informed assessment could raise suspicion for hypoxia-associated or biologically aggressive subregions within an otherwise favorable HPV-positive tumor. In this regard, such imaging findings may prompt closer multidisciplinary discussion regarding treatment intensity, closer follow-up, or early response monitoring, particularly in patients considered for treatment de-intensification protocols. Though these interpretations remain investigational, they illustrate how radiogenomic insights may complement standard imaging assessment rather than replace it.

**Figure 4:**
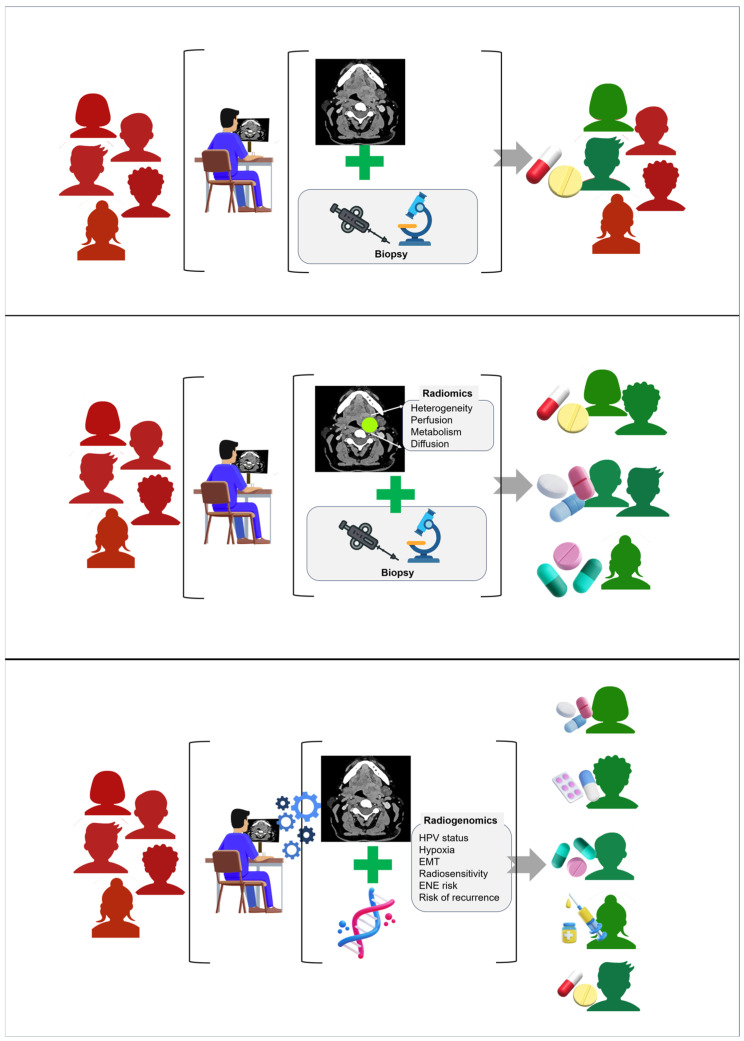
Conceptual model illustrating the progression from conventional therapeutic assignment to radiomics-guided decision-making and ultimately to non-invasive radiogenomic precision medicine. Upper panel depicts conventional treatment allocation based primarily on qualitative imaging interpretation and clinical staging. Middle panel illustrates the incorporation of quantitative radiomic phenotyping, where imaging features derived from CT, MRI or PET/CT contribute to risk stratification and therapy selection. Lower panel shows the radiogenomic stage, in which multimodal imaging features are integrated with molecular and clinical data using AI-based models to infer tumor biology and support biologically informed treatment decisions. Illustrative representation reflecting ideas discussed in Guo et al. [51].

The conceptual progression from conventional therapeutic assignment to radiomics-guided stratification and ultimately to radiogenomic prediction is illustrated in Figure 4.

## 9. Current Limitations and Pathways Toward Clinical Integration

From a dentomaxillofacial radiology perspective, OPSCC radiogenomics spans three distinct stages of maturity: what is already established, what is emerging and what remains unexplored. Established evidence demonstrates strong associations between multimodal imaging phenotypes and biological programs such as HPV-related immune activation, genomic instability, hypoxia and EMT [34,54]. Emerging work shows that AI-enhanced radiogenomics can strengthen biologically informed prediction, although reproducibility, harmonization and interpretability challenges persist. Notably, CBCT-based radiogenomics in OPSCC remains entirely unexplored, despite CBCT’s ability to capture high-resolution bone-adjacent texture patterns and soft-tissue asymmetries, its role in OPSCC radiogenomics remains exploratory and may be considered a potential area for future investigation. The absence of molecularly annotated CBCT datasets represents a clear and timely research opportunity for the dentomaxillofacial community.

Radiogenomic research in OPSCC remains constrained by important methodological and data-related limitations. The availability of imaging–genomics datasets is uneven across modalities, with most studies relying on retrospective, single-institution cohorts derived from CT, MRI or PET/CT, often without standardized multicenter harmonization. Molecular annotation is incorporated in only a subset of investigations, frequently through linkage with genomic resources such as TCGA or locally curated biobanks. Consequently, large publicly accessible imaging–genomics datasets remain scarce, and externally validated radiogenomic models are still uncommon [10,12,25,33,37,61].

These constraints contribute to substantial methodological heterogeneity and limit the generalizability of reported findings. Radiomic features are highly sensitive to variations in acquisition parameters, reconstruction algorithms and segmentation approaches, which can lead to instability across scanners and institutions. As a result, models developed in single-center datasets often demonstrate reduced performance when applied to external cohorts. Although many studies report promising predictive performance, the lack of multicenter validation and the scarcity of prospective radiogenomic investigations remain significant barriers to clinical translation [18,25,29,33,35,37,39].

Together, these limitations highlight the need for coordinated efforts to develop standardized imaging protocols, harmonized feature extraction pipelines and large multicenter datasets integrating comprehensive molecular characterization. Such advances will be essential to ensure the reproducibility, robustness and clinical applicability of radiogenomic biomarkers in OPSCC [18,29,61].

Another important limitation concerns the scarcity of prospective validation studies. Most radiogenomic investigations in OPSCC rely on retrospective imaging datasets, frequently collected for diagnostic rather than research purposes. Prospective radiogenomic trials integrating standardized imaging acquisition, molecular profiling and clinical endpoints remain rare, limiting the ability to determine whether reported imaging–genomic correlations can reliably support clinical decision-making in real-world settings [10,25,33,37].

Despite substantial progress, several barriers continue to limit the clinical adoption of radiogenomics in OPSCC. A major challenge is the pronounced variability introduced by differences in scanners, reconstruction algorithms, and acquisition protocols, which affects feature robustness and hampers reproducibility across centers. Similar concerns have been emphasized in broader radiogenomics literature, where the harmonization of imaging data is repeatedly identified as a prerequisite for model generalization [19,29,39,61].

Beyond technical considerations, the clinical deployment of AI-driven radiogenomic tools also raises regulatory, ethical and data-governance challenges. Issues related to data privacy, algorithm transparency, and regulatory approval pathways must be addressed before AI-based radiogenomic models can be integrated into routine clinical workflows. These considerations are particularly relevant for multicenter radiogenomic datasets that combine imaging, genomic and clinical information across institutions [19,29,61].

The anatomical complexity of the oropharynx further compounds segmentation difficulties, particularly when tumor boundaries, necrotic components and post-treatment changes blend into adjacent soft tissues.

Interpretability remains a further concern. Traditional radiomic models already face criticism for their limited biological transparency; AI-enhanced radiogenomics compounds this by incorporating multi-layered, non-linear representations that are difficult to trace back to specific imaging or molecular drivers. As emphasized in current AI literature, the widespread adoption of radiogenomic tools will depend on explainability frameworks that allow clinicians to understand why a model predicts a given biological signature or treatment response [61].

Promising future directions include the integration of radiogenomics with low-cost, minimally invasive molecular assays such as circulating-tumor DNA or transcriptomic surrogates, enabling biological validation in larger cohorts [19,29,70]. Habitat imaging and voxel-level radiogenomic correlation may also enhance biological interpretability by linking spatially distinct imaging subregions to specific molecular programs, a concept gaining traction in tumors with marked intratumoral heterogeneity [61].

It is important to emphasize that the inclusion of CBCT in this review is not intended to imply that CBCT-based radiogenomics in OPSCC has already achieved clinical validation. Rather, the lack of published CBCT radiomics or radiogenomics studies in OPSCC is explicitly acknowledged as an unresolved knowledge gap. In translational imaging research, delineating such gaps alongside established evidence is essential to guide future investigation. In this context, CBCT is discussed as a hypothesis-generating and pre-diagnostic modality, supported by its established capacity to depict high-resolution bone-adjacent texture patterns and incidental soft-tissue findings in dentomaxillofacial practice. Explicitly identifying CBCT as an unexplored platform helps define concrete research priorities without prematurely extrapolating clinical applicability.

Dentomaxillofacial radiologists are especially well positioned to contribute to this evolution. Their expertise in multimodal head and neck imaging, interpretation of subtle soft-tissue patterns [81] and integration of CBCT findings into clinical context provides a critical link between traditional radiologic assessment and biologically informed precision oncology. As radiogenomics matures, the role of dentomaxillofacial radiologists will increasingly change from identifying structural abnormalities to interpreting imaging phenotypes as manifestations of underlying tumor biology.

## 10. Conclusions

Radiogenomics provides a framework for investigating associations between imaging phenotypes and the underlying biology of OPSCC. Rather than viewing CT, MRI and PET/CT findings solely as structural descriptors, radiomics and radiogenomic studies have suggested that imaging phenotypes may be associated with biological processes such as immune activity, hypoxia, genomic instability and other tumor behaviors relevant to prognosis and treatment planning. However, most of these associations derive from retrospective radiomics studies and therefore require validation in larger, multicenter cohorts before clinical application can be considered.

At present, the most consistent evidence relates to associations between HPV status and imaging phenotype, whereas many other imaging–genomic relationships remain exploratory and dependent on study design, cohort characteristics and analytical methodology. CBCT may be considered a potential imaging platform within dentomaxillofacial radiology; however, its radiogenomic relevance in OPSCC has not yet been established and should be regarded as a hypothesis-generating research direction.

The concepts discussed in this review highlight several research priorities, including the development of molecularly annotated imaging datasets, harmonized acquisition protocols and prospective validation studies. Current evidence supports the biological plausibility of radiogenomic imaging signatures in OPSCC, but most proposed clinical applications remain investigational. Future studies integrating multimodal imaging, molecular profiling and standardized analytical frameworks will be necessary to determine whether radiogenomic approaches can meaningfully contribute to clinical decision-making in dentomaxillofacial radiology.

## Figures and Tables

**Table 1:** Literature selection framework used in this review.

Stage	Description	Number of Records
Identification	Database search across PubMed/MEDLINE, Scopus and Web of Science using radiomics and OPSCC-related keywords	643
Screening	Title and abstract screening to identify studies related to imaging biomarkers and radiogenomics in OPSCC	168
Eligibility	Full-text evaluation of radiomics/radiogenomics studies and biologically relevant head and neck cancer investigations	103
Inclusion	81 studies included in the narrative synthesis	81

**Table 2:** Genomic subtypes of OPSCC and their corresponding imaging phenotypes.

Molecular Subtype	Genomic Features	Clinical Profile	Imaging Characteristics	Evidence Level
HPV-positive, immune-active	PIK3CA, TRAF3; low mutational burden	Favorable prognosis; radiosensitive	Relatively homogeneous enhancement, lower entropy, cohesive ADC distributions, cystic nodal metastases	Moderate
HPV-negative, hypoxic/EMT-driven	TP53, FAT1, NOTCH1; hypoxia-related pathways	Poor treatment response; higher recurrence risk	Greater heterogeneity, necrotic components, irregular margins, low-ADC regions	Moderate
HPV-negative, proliferative/stemness-associated	TERT promoter alterations; genomic instability	Aggressive behavior; early recurrence	Increased metabolic heterogeneity on PET/CT, pronounced glycolytic gradients	Limited
HPV-positive, lower-risk metabolic profile	Immune-rich microenvironment	Most favorable survival	Smoother imaging patterns, lower metabolic activity, reduced textural complexity	Limited

Note: ADC, apparent diffusion coefficient; CNV, copy-number variation; DWI, diffusion-weighted imaging; EMT, epithelial–mesenchymal transition; FDG, fluorodeoxyglucose; MTV, metabolic tumor volume; PET, positron emission tomography; PIK3CA, phosphatidylinositol-4,5-bisphosphate 3-kinase catalytic subunit alpha; RT, radiotherapy; TERT, telomerase reverse transcriptase; TLG, total lesion glycolysis; TRAF3, TNF receptor-associated factor 3.

**Table 3:** Biological programs linked to radiogenomic signatures in OPSCC.

Biological Program	Genomic Drivers	Radiomic Expression	Prognostic Interpretation	Evidence Type
Genomic Stability	PIK3CA	Associated with lower entropy	Favorable	Retrospective radiomics
Genomic Instability	TP53	Associated with increased entropy	Poor prognosis	Retrospective radiomics
Hypoxia	HIF1A	Associated with low-ADC regions	Recurrence risk	Radiomics/PET
EMT	SNAI2	Irregular margins	Nodal spread	Radiomics
Metabolic	TERT	High MTV/TLG	Poor prognosis	PET radiomics

Note: ADC, apparent diffusion coefficient; EMT, epithelial–mesenchymal transition; ENE, extranodal extension; FAT1, FAT atypical cadherin 1; HIF1A, hypoxia-inducible factor 1-alpha; mTOR, mechanistic target of rapamycin; MTV, metabolic tumor volume; NOTCH1, neurogenic locus notch homolog protein 1; PET, positron emission tomography; PIK3CA, phosphatidylinositol-4,5-bisphosphate 3-kinase catalytic subunit alpha; PTEN, phosphatase and tensin homolog; SNAI2, snail family transcriptional repressor 2; TLG, total lesion glycolysis; TRAF3, TNF receptor–associated factor 3; TP53, tumor protein p53; TWIST1, twist family bHLH transcription factor 1; VEGFA, vascular endothelial growth factor A; ZEB1, zinc finger E-box binding homeobox 1.

**Table 4:** Overview of radiomics and radiogenomics studies relevant to OPSCC.

Category	Number of Studies	References
CT-based radiomics studies	4	[25,33,34,35]
MRI-based radiomics studies	3	[22,23,32]
PET/CT radiomics studies	2	[12,36]
Multimodal radiogenomic analyses	4	[10,18,29,35]
Habitat-based or spatial radiomics approaches	1	[13]
Machine learning–based models	8	[10,12,25,33,35,37,38,39]
Deep learning approaches	2	[40,41]
Radiomics statistical modeling studies	5	[22,23,33,37,42]
HPV status prediction	4	[14,25,35,39]
Hypoxia/microenvironment signatures	2	[11,42]
Immune microenvironment correlations	2	[13,43]
Prognosis/survival prediction	5	[10,12,33,35,37]
Treatment response prediction	3	[12,33,44]
Nodal metastasis prediction	2	[13,15]

Note: CBCT, cone-beam computed tomography; CT, computed tomography; HPV, human papillomavirus; PET, positron emission tomography. This table includes studies specifically conducted in OPSCC as well as selected head and neck cancer studies that provide methodological or biological context relevant to OPSCC radiogenomics.

**Table 5:** Imaging modalities and their clinical applications in OPSCC with corresponding levels of evidence.

Imaging Modality	Typical Visual Features	Clinical Application	Evidence Level
CT	Enhancement, necrosis, margins	HPV inference, ENE detection	Moderate
MRI	Diffusion, perfusion	Response assessment	Moderate
PET/CT	Metabolic gradients	Prognosis	Moderate
CBCT	Bone + incidental findings	Detection/referral	Limited/exploratory

Note: CBCT, cone-beam computed tomography; CT, computed tomography; ENE, extranodal extension; HPV, human papillomavirus; PET, positron emission tomography.

**Table 6:** AI and machine learning integration in OPSCC Radiogenomics.

AI Strategy	Data Source	Biological Insight Learned	Phenotypes Captured	Clinical Utility	Evidence Status
Deep Learning	CT/MRI/PET image patches	Immune signatures, EMT patterns	Texture probability maps	HPV status prediction	Emerging
Multimodal Fusion Models	Imaging + genomic + clinical data	Hypoxia and oxidative stress pathways	Perfusion–diffusion integration	Radiosensitivity prediction	Emerging
Radiomics + Machine Learning	Radiomic feature matrices + clinical variables	Stromal and microenvironment programs	Entropy distribution and heterogeneity	Extranodal extension (ENE) prediction	Moderate evidence
Delta-Radiomics	Serial imaging datasets	Dynamic treatment adaptation signals	Temporal changes in ADC/metabolic activity	Early non-responder identification	Conceptual/exploratory
Graph Neural Networks (GNN)	Spatially structured tumor regions	Tumor–microenvironment spatial interactions	Propagation of heterogeneity across regions	Metastatic spread modeling	Conceptual (limited evidence)
Explainable AI	Model attribution maps	Pathway–phenotype alignment	Saliency maps/heatmaps	Multidisciplinary interpretability	Emerging

## Data Availability

No new data were created or analyzed in this study. Data sharing is not applicable to this article.

## Supplementary Materials

The following supporting information can be downloaded at: https://www.mdpi.com/article/10.3390/medsci14020174/s1, Table S1. Characteristics of representative studies informing radiomics and radiogenomics in OPSCC.

## References

[1] Ang K.K., Harris J., Wheeler R., Weber R., Rosenthal D.I., Nguyen-Tân P.F., Westra W.H., Chung C.H., Jordan R.C., Lu C. (2010). Human Papillomavirus and Survival of Patients with Oropharyngeal Cancer. N. Engl. J. Med..

[2] Lechner M., Liu J., Masterson L., Fenton T.R. (2022). HPV-associated oropharyngeal cancer: Epidemiology, molecular biology and clinical management. Nat. Rev. Clin. Oncol..

[3] Tsai C.-C., Hsu Y.-C., Chu T.-Y., Hsu P.-C., Kuo C.-Y. (2025). Immune Evasion in Head and Neck Squamous Cell Carcinoma: Roles of Cancer-Associated Fibroblasts, Immune Checkpoints, and TP53 Mutations in the Tumor Microenvironment. Cancers.

[4] Ko H.C., Harari P.M., Sacotte R.M., Chen S., Wieland A.M., Yu M., Baschnagel A.M., Bruce J.Y., Kimple R.J., Witek M.E. (2017). Prognostic implications of human papillomavirus status for patients with non-oropharyngeal head and neck squamous cell carcinomas. J. Cancer Res. Clin. Oncol..

[5] O’Sullivan B., Huang S.H., Siu L.L., Waldron J., Zhao H., Perez-Ordonez B., Weinreb I., Kim J., Ringash J., Bayley A. (2013). Deintensification Candidate Subgroups in Human Papillomavirus–Related Oropharyngeal Cancer According to Minimal Risk of Distant Metastasis. J. Clin. Oncol..

[6] Goldenberg D., Begum S., Westra W.H., Khan Z., Sciubba J., Pai S.I., Califano J.A., Tufano R.P., Koch W.M. (2008). Cystic lymph node metastasis in patients with head and neck cancer: An HPV-associated phenomenon. Head Neck.

[7] Chan M.W., Yu E., Bartlett E., O’Sullivan B., Su J., Waldron J., Ringash J., Bratman S.V., Chen Y.A., Irish J. (2017). Morphologic and topographic radiologic features of human papillomavirus-related and –unrelated oropharyngeal carcinoma. Head Neck.

[8] Cantrell S.C., Peck B.W., Li G., Wei Q., Sturgis E.M., Ginsberg L.E. (2013). Differences in Imaging Characteristics of HPV-Positive and HPV-Negative Oropharyngeal Cancers: A Blinded Matched-Pair Analysis. AJNR Am. J. Neuroradiol..

[9] Ong Y.H., Zheng W., Khong P.L., Ni Q. (2024). Application of radiogenomics in head and neck cancer: A new tool toward diagnosis and therapy. Interdiscip. Radiol..

[10] Rabasco Meneghetti A., Zwanenburg A., Linge A., Lohaus F., Grosser M., Baretton G.B., Kalinauskaite G., Tinhofer I., Guberina M., Stuschke M. (2022). Integrated radiogenomics analyses allow for subtype classification and improved outcome prognosis of patients with locally advanced HNSCC. Sci. Rep..

[11] Huang M., Law H.K.W., Tam S.Y. (2025). Use of Radiomics in Characterizing Tumor Hypoxia. Int. J. Mol. Sci..

[12] Spielvogel C.P., Stoiber S., Papp L., Krajnc D., Grahovac M., Gurnhofer E., Trachtova K., Bystry V., Leisser A., Jank B. (2023). Radiogenomic markers enable risk stratification and inference of mutational pathway states in head and neck cancer. Eur. J. Nucl. Med. Mol. Imaging.

[13] Chen X., Jiang H., Pan M., Feng C., Li Y., Chen L., Luo Y., Liu L., Peng J., Hu G. (2025). Habitat radiomics predicts occult lymph node metastasis and uncovers immune microenvironment of head and neck cancer. J. Transl. Med..

[14] Ansari G., Mirza-Aghazadeh-Attari M., Mosier K.M., Fakhry C., Yousem D.M. (2024). Radiomics Features in Predicting Human Papillomavirus Status in Oropharyngeal Squamous Cell Carcinoma: A Systematic Review, Quality Appraisal, and Meta-Analysis. Diagnostics.

[15] Lu S., Ling H., Chen J., Tan L., Gao Y., Li H., Tan P., Huang D., Zhang X., Liu Y. (2022). MRI-based radiomics analysis for preoperative evaluation of lymph node metastasis in hypopharyngeal squamous cell carcinoma. Front. Oncol..

[16] Chi A.C., Day T.A., Neville B.W. (2015). Oral cavity and oropharyngeal squamous cell carcinoma—An update. CA Cancer J. Clin..

[17] Weatherspoon D.J., Chattopadhyay A., Boroumand S., Garcia I. (2015). Oral cavity and oropharyngeal cancer incidence trends and disparities in the United States: 2000–2010. Cancer Epidemiol..

[18] Bruixola G., Remacha E., Jiménez-Pastor A., Dualde D., Viala A., Montón J.V., Ibarrola-Villava M., Alberich-Bayarri Á., Cervantes A. (2021). Radiomics and radiogenomics in head and neck squamous cell carcinoma: Potential contribution to patient management and challenges. Cancer Treat. Rev..

[19] Saxena S., Jena B., Gupta N., Das S., Sarmah D., Bhattacharya P., Nath T., Paul S., Fouda M.M., Kalra M. (2022). Role of Artificial Intelligence in Radiogenomics for Cancers in the Era of Precision Medicine. Cancers.

[20] Gillison M.L., Chaturvedi A.K., Anderson W.F., Fakhry C. (2015). Epidemiology of Human Papillomavirus-Positive Head and Neck Squamous Cell Carcinoma. J. Clin. Oncol..

[21] Khoo A., Boyer M., Jafri Z., Makeham T., Pham T., Khachigian L.M., Floros P., Dowling E., Fedder K., Shonka D. (2024). Human Papilloma Virus Positive Oropharyngeal Squamous Cell Carcinoma and the Immune System: Pathogenesis, Immunotherapy and Future Perspectives. Int. J. Mol. Sci..

[22] Lenoir V., Delattre B.M.A., M’Rad Y., De Vito C., De Perrot T., Becker M. (2022). Diffusion-Weighted Imaging to Assess HPV-Positive versus HPV-Negative Oropharyngeal Squamous Cell Carcinoma: The Importance of b-Values. AJNR Am. J. Neuroradiol..

[23] Bollen H., Dok R., De Keyzer F., Deschuymer S., Laenen A., Devos J., Vandecaveye V., Nuyts S. (2024). Diffusion-Weighted MRI and Human Papillomavirus (HPV) Status in Oropharyngeal Cancer. Cancers.

[24] Chen L.L., Lauwers I., Verduijn G., Philippens M., Gahrmann R., Capala M.E., Petit S. (2024). MRI for Differentiation between HPV-Positive and HPV-Negative Oropharyngeal Squamous Cell Carcinoma: A Systematic Review. Cancers.

[25] Song B., Yang K., Garneau J., Lu C., Li L., Lee J., Stock S., Braman N.M., Koyuncu C.F., Toro P. (2021). Radiomic Features Associated With HPV Status on Pretreatment Computed Tomography in Oropharyngeal Squamous Cell Carcinoma Inform Clinical Prognosis. Front. Oncol..

[26] Alsahafi E., Begg K., Amelio I., Raulf N., Lucarelli P., Sauter T., Tavassoli M. (2019). Clinical update on head and neck cancer: Molecular biology and ongoing challenges. Cell Death Dis..

[27] Zhang Y., Koneva L.A., Virani S., Arthur A.E., Virani A., Hall P.B., Warden C.D., Carey T.E., Chepeha D.B., Prince M.E. (2016). Subtypes of HPV-Positive Head and Neck Cancers Are Associated with HPV Characteristics, Copy Number Alterations, PIK3CA Mutation, and Pathway Signatures. Clin. Cancer Res..

[28] Leemans C.R., Snijders P.J.F., Brakenhoff R.H. (2018). The molecular landscape of head and neck cancer. Nat. Rev. Cancer.

[29] Mendes Serrão E., Klug M., Moloney B.M., Jhaveri A., Lo Gullo R., Pinker K., Luker G., Haider M.A., Shinagare A.B., Liu X. (2023). Current Status of Cancer Genomics and Imaging Phenotypes: What Radiologists Need to Know. Radiol. Imaging Cancer.

[30] Qin T., Li S., Henry L.E., Liu S., Sartor M.A. (2021). Molecular Tumor Subtypes of HPV-Positive Head and Neck Cancers: Biological Characteristics and Implications for Clinical Outcomes. Cancers.

[31] Farah C.S. (2021). Molecular landscape of head and neck cancer and implications for therapy. Ann. Transl. Med..

[32] Dong H., Shu X., Xu Q., Zhu C., Kaufmann A.M., Zheng Z.-M., Albers A.E., Qian X. (2021). Current Status of Human Papillomavirus-Related Head and Neck Cancer: From Viral Genome to Patient Care. Virol. Sin..

[33] Parmar C., Grossmann P., Rietveld D., Rietbergen M.M., Lambin P., Aerts H.J.W.L. (2015). Radiomic Machine-Learning Classifiers for Prognostic Biomarkers of Head and Neck Cancer. Front. Oncol..

[34] Aerts H.J.W.L., Velazquez E.R., Leijenaar R.T.H., Parmar C., Grossmann P., Carvalho S., Bussink J., Monshouwer R., Haibe-Kains B., Rietveld D. (2014). Decoding tumour phenotype by noninvasive imaging using a quantitative radiomics approach. Nat. Commun..

[35] Katsoulakis E., Yu Y., Apte A.P., Leeman J.E., Katabi N., Morris L., Deasy J.O., Chan T.A., Lee N.Y., Riaz N. (2020). Radiomic analysis identifies tumor subtypes associated with distinct molecular and microenvironmental factors in head and neck squamous cell carcinoma. Oral Oncol..

[36] Hatt M., Tixier F., Pierce L., Kinahan P.E., Le Rest C.C., Visvikis D. (2017). Characterization of PET/CT images using texture analysis: The past, the present… any future?. Eur. J. Nucl. Med. Mol. Imaging.

[37] Kuno H., Qureshi M.M., Chapman M.N., Li B., Andreu-Arasa V.C., Onoue K., Truong M.T., Sakai O. (2017). CT Texture Analysis Potentially Predicts Local Failure in Head and Neck Squamous Cell Carcinoma Treated with Chemoradiotherapy. AJNR Am. J. Neuroradiol..

[38] Zhu Y., Mohamed A.S.R., Lai S.Y., Yang S., Kanwar A., Wei L., Kamal M., Sengupta S., Elhalawani H., Skinner H. (2019). Imaging-Genomic Study of Head and Neck Squamous Cell Carcinoma: Associations Between Radiomic Phenotypes and Genomic Mechanisms via Integration of The Cancer Genome Atlas and The Cancer Imaging Archive. JCO Clin. Cancer Inform..

[39] Reiazi R., Arrowsmith C., Welch M., Abbas-Aghababazadeh F., Eeles C., Tadic T., Hope A.J., Bratman S.V., Haibe-Kains B. (2021). Prediction of Human Papillomavirus (HPV) Association of Oropharyngeal Cancer (OPC) Using Radiomics: The Impact of the Variation of CT Scanner. Cancers.

[40] Tian R., Hou F., Zhang H., Yu G., Yang P., Li J., Yuan T., Chen X., Chen Y., Hao Y. (2025). Multimodal fusion model for prognostic prediction and radiotherapy response assessment in head and neck squamous cell carcinoma. npj Digit. Med..

[41] Ardila D., Kiraly A.P., Bharadwaj S., Choi B., Reicher J.J., Peng L., Tse D., Etemadi M., Ye W., Corrado G. (2019). End-to-end lung cancer screening with three-dimensional deep learning on low-dose chest computed tomography. Nat. Med..

[42] Huang W., Li N., Zhu S., Zhang Y., Song X., Zhang B., Yan H., Tian J., Wang K., Zhang S. (2025). Imaging hypoxia for head and neck cancer: Current status, challenges, and prospects. Theranostics.

[43] Yin J., Xu L., Wang S., Zhang L., Zhang Y., Zhai Z., Zeng P., Grzegorzek M., Jiang T. (2024). Integrating immune multi-omics and machine learning to improve prognosis, immune landscape, and sensitivity to first- and second-line treatments for head and neck squamous cell carcinoma. Sci. Rep..

[44] Tixier F., Le Rest C.C., Hatt M., Albarghach N., Pradier O., Metges J.-P., Corcos L., Visvikis D. (2011). Intratumor Heterogeneity Characterized by Textural Features on Baseline ^18^F-FDG PET Images Predicts Response to Concomitant Radiochemotherapy in Esophageal Cancer. J. Nucl. Med..

[45] Price J.B., Thaw K.L., Tyndall D.A., Ludlow J.B., Padilla R.J. (2012). Incidental findings from cone beam computed tomography of the maxillofacial region: A descriptive retrospective study. Clin. Oral Implants Res..

[46] Barghan S., Tahmasbi Arashlow M., Nair M.K. (2016). Incidental Findings on Cone Beam Computed Tomography Studies outside of the Maxillofacial Skeleton. Int. J. Dent..

[47] Aflah K.A., Yohana W., Oscandar F. (2022). Volumetric measurement of the tongue and oral cavity with cone-beam computed tomography: A systematic review. Imaging Sci. Dent..

[48] Paniagua B., Ruellas A.C., Benavides E., Marron S., Woldford L., Cevidanes L. (2015). Validation of CBCT for the computation of textural biomarkers. Proc. SPIE Int. Soc. Opt. Eng..

[49] Gonçalves B.C., De Araújo E.C., Nussi A.D., Bechara N., Sarmento D., Oliveira M.S., Santamaria M.P., Costa A.L.F., Lopes S. (2020). Texture analysis of cone-beam computed tomography images assists the detection of furcal lesion. J. Periodontol..

[50] De Rosa C.S., Bergamini M.L., Palmieri M., Sarmento D.J.D.S., De Carvalho M.O., Ricardo A.L.F., Hasseus B., Jonasson P., Braz-Silva P.H., Ferreira Costa A.L. (2020). Differentiation of periapical granuloma from radicular cyst using cone beam computed tomography images texture analysis. Heliyon.

[51] Park S., Jeon S.-J., Yeom H.-G., Seo M.-S. (2024). Differential diagnosis of cemento-osseous dysplasia and periapical cyst using texture analysis of CBCT. BMC Oral Health.

[52] İçöz D., Çetin B., Dinç K. (2025). Application of radiomics features in differential diagnosis of odontogenic cysts. Dentomaxillofac. Radiol..

[53] Queiroz P.M., Fardim K.C., Costa A.L.F., Matheus R.A., Lopes S.L.P.C. (2023). Texture analysis in cone-beam computed tomographic images of medication-related osteonecrosis of the jaw. Imaging Sci. Dent..

[54] Zwanenburg A., Vallières M., Abdalah M.A., Aerts H.J.W.L., Andrearczyk V., Apte A., Ashrafinia S., Bakas S., Beukinga R.J., Boellaard R. (2020). The Image Biomarker Standardization Initiative: Standardized Quantitative Radiomics for High-Throughput Image-based Phenotyping. Radiology.

[55] Park B.W., Kim J.K., Heo C., Park K.J. (2020). Reliability of CT radiomic features reflecting tumour heterogeneity according to image quality and image processing parameters. Sci. Rep..

[56] Bianchi J., Gonçalves J.R., Ruellas A.C.D.O., Vimort J.-B., Yatabe M., Paniagua B., Hernandez P., Benavides E., Soki F.N., Cevidanes L.H.S. (2019). Software comparison to analyze bone radiomics from high resolution CBCT scans of mandibular condyles. Dentomaxillofac. Radiol..

[57] Flaiban E., Orhan K., Gonçalves B.C., Lopes S.L.P.D.C., Costa A.L.F. (2025). Radiomics in Action: Multimodal Synergies for Imaging Biomarkers. Bioengineering.

[58] Delgadillo R., Ford J.C., Abramowitz M.C., Dal Pra A., Pollack A., Stoyanova R. (2020). The role of radiomics in prostate cancer radiotherapy. Strahlenther. Onkol..

[59] Ma Y., Liu A., O’Connell A.M., Zhu Y., Li H., Han P., Yin L., Lu H., Ye Z. (2021). Contrast-enhanced cone beam breast CT features of breast cancers: Correlation with immunohistochemical receptors and molecular subtypes. Eur. Radiol..

[60] Lo Gullo R., Daimiel I., Morris E.A., Pinker K. (2020). Combining molecular and imaging metrics in cancer: Radiogenomics. Insights Imaging.

[61] Guo Y., Li T., Gong B., Hu Y., Wang S., Yang L., Zheng C. (2025). From Images to Genes: Radiogenomics Based on Artificial Intelligence to Achieve Non-Invasive Precision Medicine in Cancer Patients. Adv. Sci..

[62] Secerov-Ermenc A., Peterlin P., Anderluh F., But-Hadzic J., Jeromen-Peressutti A., Velenik V., Segedin B. (2024). Inter-observer variation in gross tumour volume delineation of oesophageal cancer on MR, CT and PET/CT. Radiol. Oncol..

[63] Katsura K., Tanabe S., Nakano H., Sakai M., Ohta A., Kaidu M., Soga M., Kobayashi T., Takamura M., Hayashi T. (2023). The Relationship between the Contouring Time of the Metal Artifacts Area and Metal Artifacts in Head and Neck Radiotherapy. Tomography.

[64] Walter A., Hoegen-Saßmannshausen P., Stanic G., Rodrigues J.P., Adeberg S., Jäkel O., Frank M., Giske K. (2024). Segmentation of 71 Anatomical Structures Necessary for the Evaluation of Guideline-Conforming Clinical Target Volumes in Head and Neck Cancers. Cancers.

[65] Ren J., Teuwen J., Nijkamp J., Rasmussen M., Gouw Z., Grau Eriksen J., Sonke J.-J., Korreman S. (2024). Enhancing the reliability of deep learning-based head and neck tumour segmentation using uncertainty estimation with multi-modal images. Phys. Med. Biol..

[66] Gillies R.J., Kinahan P.E., Hricak H. (2016). Radiomics: Images Are More than Pictures, They Are Data. Radiology.

[67] Kumar V., Gu Y., Basu S., Berglund A., Eschrich S.A., Schabath M.B., Forster K., Aerts H.J., Dekker A., Fenstermacher D. (2012). Radiomics: The process and the challenges. Magn. Reson. Imaging.

[68] Lambin P., Rios-Velazquez E., Leijenaar R., Carvalho S., Van Stiphout R.G.P.M., Granton P., Zegers C.M.L., Gillies R., Boellard R., Dekker A. (2012). Radiomics: Extracting more information from medical images using advanced feature analysis. Eur. J. Cancer.

[69] He X., Chen Z., Gao Y., Wang W., You M. (2023). Reproducibility and location-stability of radiomic features derived from cone-beam computed tomography: A phantom study. Dentomaxillofac. Radiol..

[70] Fave X., Mackin D., Yang J., Zhang J., Fried D., Balter P., Followill D., Gomez D., Kyle Jones A., Stingo F. (2015). Can radiomics features be reproducibly measured from CBCT images for patients with non-small cell lung cancer?. Med. Phys..

[71] Saltz J., Gupta R., Hou L., Kurc T., Singh P., Nguyen V., Samaras D., Shroyer K.R., Zhao T., Batiste R. (2018). Spatial Organization and Molecular Correlation of Tumor-Infiltrating Lymphocytes Using Deep Learning on Pathology Images. Cell Rep..

[72] Kather J.N., Pearson A.T., Halama N., Jäger D., Krause J., Loosen S.H., Marx A., Boor P., Tacke F., Neumann U.P. (2019). Deep learning can predict microsatellite instability directly from histology in gastrointestinal cancer. Nat. Med..

[73] Echle A., Rindtorff N.T., Brinker T.J., Luedde T., Pearson A.T., Kather J.N. (2021). Deep learning in cancer pathology: A new generation of clinical biomarkers. Br. J. Cancer.

[74] Trivizakis E., Papadakis G., Souglakos I., Papanikolaou N., Koumakis L., Spandidos D., Tsatsakis A., Karantanas A., Marias K. (2020). Artificial intelligence radiogenomics for advancing precision and effectiveness in oncologic care (Review). Int. J. Oncol..

[75] Ismail M., Hanifa M.A.M., Mahidin E.I.M., Manan H.A., Yahya N. (2024). Cone beam computed tomography (CBCT) and megavoltage computed tomography (MVCT)-based radiomics in head and neck cancers: A systematic review and radiomics quality score assessment. Quant. Imaging Med. Surg..

[76] van der Velden B.H.M., Kuijf H.J., Gilhuijs K.G.A., Viergever M.A. (2022). Explainable artificial intelligence (XAI) in deep learning-based medical image analysis. Med. Image Anal..

[77] Neri E., Aghakhanyan G., Zerunian M., Gandolfo N., Grassi R., Miele V., Giovagnoni A., Laghi A. (2023). Explainable AI in radiology: A white paper of the Italian Society of Medical and Interventional Radiology. Radiol. Med..

[78] Bhatia A., Burtness B. (2015). Human Papillomavirus-Associated Oropharyngeal Cancer: Defining Risk Groups and Clinical Trials. J. Clin. Oncol..

[79] Chakrabarty N., Mahajan A. (2024). Radiological extranodal extension in head and neck cancers: Current evidence and challenges in imaging detection and prognostic impact. BJR Open.

[80] Caprini E., D’Agnese G., Brennan P.A., Rahimi S. (2023). Human papillomavirus–related oropharyngeal squamous cell carcinoma and radiomics: A new era?. J. Oral Pathol..

[81] Santos G.N.M., Da Silva H.E.C., Ossege F.E.L., Figueiredo P.T.D.S., Melo N.D.S., Stefani C.M., Leite A.F. (2023). Radiomics in bone pathology of the jaws. Dentomaxillofac. Radiol..

