# Supplementary material for: Multimodal Radiogenomic Imaging in Oropharyngeal Squamous Cell Carcinoma: Implications for Dentomaxillofacial Radiology
Source: Med Sci (Basel). 2026 Mar 31;14(2):174. doi: 10.3390/medsci14020174 (PMC13108179; doi:10.3390/medsci14020174)
Supplement: Supplementary file 1 [file medsci-14-00174-s001.zip › medsci-4170293-supplementary.pdf]

Supplementary Table S1. Characteristics of representative studies informing radiomics and radiogenomics in OPSCC

Reference	Imaging Modality	Cohort Type	Sample Size	Study Type / Analytical Approach	Primary Outcome
Ansari et al., 2024 [14]	CT / MRI	Systematic review	NA	Systematic review of radiomics studies	HPV status prediction
Song et al., 2021 [25]	CT	Institutional	153	Radiomics + ML	HPV status
Rabasco Meneghetti et al., 2022 [10]	CT	Institutional	116	Radiogenomics + ML	Survival prediction / subtype classification
Spielvogel et al., 2023 [12]	PET/CT	Institutional	90	Radiogenomics + ML	Risk stratification / inference of mutational pathway states
Lenoir et al., 2022 [22]	MRI (DWI/ADC)	Institutional	80	Imaging phenotype / diffusion analysis	HPV differentiation
Bollen et al., 2024 [23]	MRI (DWI)	Institutional	94	Imaging phenotype / longitudinal MRI analysis	HPV-related diffusion differences / treatment-related changes
Chen et al., 2025 [13]	Multimodal / habitat imaging	Institutional	NR	Habitat radiomics	Occult nodal metastasis / immune microenvironment
Chan et al., 2017 [7]	CT	Institutional	111	Imaging phenotype analysis	HPV-related imaging features
Cantrell et al., 2013 [8]	CT	Institutional	98	Imaging phenotype analysis	HPV differentiation
Qin et al., 2021 [30]	Genomic / molecular	Institutional / translational	120	Molecular subtype characterization	HPV-positive molecular subtypes
Ong et al., 2024 [9]	CT / MRI / PET	Review	NA	Radiogenomics review	Clinical applications in head and neck cancer

Alsahafi et al., 2019 [26]	Multimodal	Review	NA	Molecular biology review	Tumor biology and imaging-related molecular context
Lechner et al., 2022 [2]	Multimodal	Review	NA	Molecular oncology review	HPV biology and clinical implications
Huang et al., 2025 [11]	Multimodal imaging	Review	NA	Imaging review	Hypoxia imaging

Note: ADC, Apparent Diffusion Coefficient; CT, Computed Tomography; DWI, Diffusion-Weighted Imaging; ML, Machine learning; Magnetic Resonance Imaging, MRI; NA, Not Applicable; PET/CT, Positron Emission Tomography/Computed Tomography. This table includes not only radiomics and radiogenomics studies, but also selected molecular and imaging studies that provide biological, or methodological context for interpreting radiogenomic associations in OPSCC.
